# shinyseg: a web application for flexible cosegregation and sensitivity analysis

**DOI:** 10.1093/bioinformatics/btae201

**Published:** 2024-04-10

**Authors:** Christian Carrizosa, Dag E Undlien, Magnus D Vigeland

**Affiliations:** Department of Medical Genetics, Oslo University Hospital and University of Oslo, 0424 Oslo, Norway; Department of Medical Genetics, Oslo University Hospital and University of Oslo, 0424 Oslo, Norway; Department of Forensic Sciences, Oslo University Hospital, 0424 Oslo, Norway

## Abstract

**Motivation:**

Cosegregation analysis is a powerful tool for identifying pathogenic genetic variants, but its implementation remains challenging. Existing software is either limited in scope or too demanding for many end users. Moreover, current solutions lack methods for assessing the robustness of cosegregation evidence, which is important due to its reliance on uncertain estimates.

**Results:**

We present shinyseg, a comprehensive web application for clinical cosegregation analysis. Our app streamlines penetrance specification based on either liability classes or epidemiological data such as risks, hazard ratios, and age of onset distribution. In addition, it incorporates sensitivity analyses to assess the robustness of cosegregation evidence, and offers support in clinical interpretation.

**Availability and implementation:**

The shinyseg app is freely available at https://chrcarrizosa.shinyapps.io/shinyseg, with documentation and complete R source code on https://chrcarrizosa.github.io/shinyseg and https://github.com/chrcarrizosa/shinyseg.

## 1 Introduction

Cosegregation analysis is a powerful tool for identifying disease-associated genetic variants (Woodbury-Smith *et al.* 2017, Caputo *et al.* 2021, Cini *et al.* 2021, Ratajska *et al.* 2023). This method uses family data to assess if the segregation of a variant aligns with the inheritance of the disease in a pedigree, and it is particularly useful for identifying rare high-risk mutations. Free from the biases of population-based designs, cosegregation represents a separate evidence source in the American College of Medical Genetics and Genomics and Association of Molecular Pathology (ACMG-AMP) guidelines for clinical variant interpretation (Richards *et al.* 2015). However, its quantitative implementation (Petersen *et al.* 1998, Thompson *et al.* 2003, Mohammadi *et al.* 2009) is technically challenging, which has resulted in the widespread use of less rigorous alternatives (Booth *et al.* 2019, Perrino *et al.* 2020, Stankute *et al.* 2022).

For many end users in clinical genetics, the main hurdle to cosegregation analysis is its reliance on specialised linkage software (Lathrop and Lalouel 1984, Schäffer *et al.* 1994). Following Richards *et al.* (2015) there have been various efforts to improve its accessibility, but these often entail a trade-off in flexibility. For instance, the meiosis-counting method of Jarvik and Browning (2016) works well in ideal examples with complete penetrance and no phenocopies, but is less useful otherwise. Similarly, the R package CoSeg (Ranola and Shirts 2016) is limited to a single autosomal dominant phenotype. Finally, the liability class approach of COOL (web server, Belman *et al.* 2020) and also segregatr (R package, Ratajska *et al.* 2023) allows multiple phenotypes and more advanced penetrance models, but is impractical unless one employs COOL's built-in cancer estimates. A detailed software comparison is included in Supplementary Section S1.

A significant limitation of current cosegregation software is the lack of sensitivity analyses. Each of the tools mentioned above outputs some likelihood-based statistic conditional on specified parameters (e.g. variant penetrances), yet reliable estimates for these are often unavailable. Ensuring the robustness of qualitative findings is vital since these can potentially impact a variant's clinical actionability. Regrettably, this is rarely done nor discussed in clinical applications, where cosegregation scores are typically taken at face value even when based on dubious penetrance parameters.

To address these shortcomings, we introduce the web application shinyseg for clinical cosegregation analysis. This offers a parametric specification of the penetrance based on simple, explicit assumptions, and allows for sensitivity analysis to assess the robustness of the results. Additionally, it retains complete flexibility for advanced users. We demonstrate its utility with the practical example presented in this article.

## 2 Implementation

shinyseg is an R-based tool built with Shiny (Chang *et al.* 2024) and rhandsontable (Owen 2021). Pedigree data are handled by the pedsuite packages (Vigeland 2021), including segregatr performing the cosegregation calculations (Ratajska *et al.* 2023). The app features tips and notifications to ensure a smooth workflow, and all analyses may be stored, shared and retrieved by means of a tailor-made HTML report format.

### 2.1 Data input

Families can be added by uploading files in standard ped format, e.g. created with QuickPed (Vigeland 2022), or chosen from a dropdown list of basic cases. Additional information is entered in a table, including each member's phenotype, genotype, proband status, and age (of disease onset or censoring), all visualized in real-time on the pedigree plot.

### 2.2 Model parameters

The default variant frequency is 0.001, but it can be adjusted as needed. The inheritance pattern is made of two choices regarding chromosome (autosomal, X-linked) and dominance (dominant, recessive, incomplete). Penetrance specification may be done in two ways.

- *Relative risk.* We introduce a parametric version of COOL's (Belman *et al.* 2020) approach based on: (i) the baseline (phenocopy) lifetime risk and age of onset, and (ii) the hazard ratios over age. The user may enter these parameters directly, or provide incidence data from which optimal parameters are inferred by the program. Variant penetrances are automatically computed following a survival model (see Supplementary Section S2 for details).
- *Liability classes.* Penetrance values for each class may be written interactively or uploaded from a suitable file.

### 2.3 Cosegregation evidence

With all necessary information in place, shinyseg computes the full-likelihood Bayes factor (FLB) for the variant's pathogenicity (Thompson *et al.* 2003). We chose this metric over the cosegregation likelihood ratio (Mohammadi *et al.* 2009) because of its greater flexibility, not being limited to a single introduction of the variant. A key for translating the cosegregation evidence into the ACMG-AMP framework is provided, using Jarvik and Browning's (2016) thresholds. Furthermore, robustness of the results can be assessed with contour plots of the FLB when varying the analysis parameters.

## 3 Results

**Figure 1.**
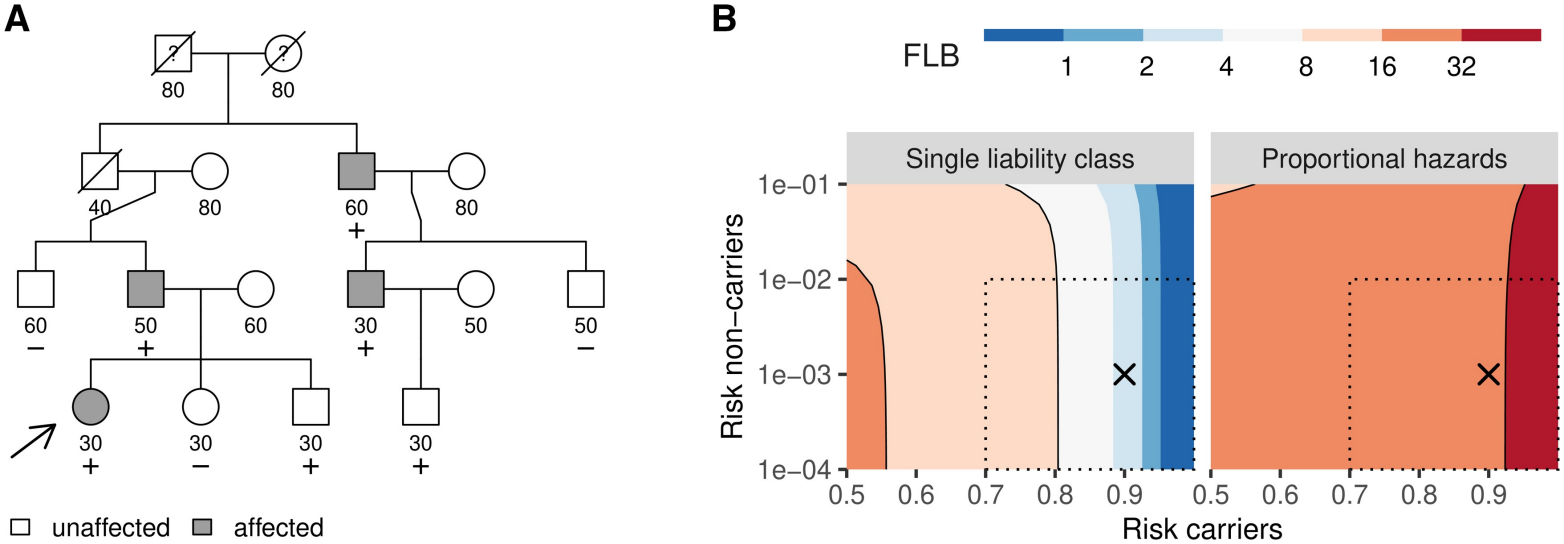
(A) Medical pedigree showing phenotype (empty: unaffected; filled: affected; question mark: unknown), age (onset or censoring) and carriership status (+: carrier; –: non-carrier). Crossed individuals are deceased; the arrow indicates the proband. (B) Sensitivity analysis displaying contours of the FLB as a function of the risk in carriers and non-carriers under two penetrance models. The crosses indicate the main results, while the dotted lines enclose a region of reasonable parameter values.

We showcase shinyseg by analyzing the pedigree in Fig. 1A. This family is based on a real case where four members affected by a rare connective tissue disorder (onset: 30–60 years) were found to share a rare autosomal variant. Among the five unaffected individuals tested, two from the youngest generation were also carriers. Moreover, the proband's grandfather is likely an obligate carrier, but he remained asymptomatic until his death in his 40s. Penetrance estimates are not available. Henceforth, we will assume complete dominance and a population variant frequency of 0.001.

A naïve segregation analysis in this family could entail setting a single risk parameter for non-carriers (phenocopy rate) and another for carriers (penetrance). Conservative values of 0.001 and 0.90, respectively, yield an FLB of 3.2, which is inconclusive evidence for the variant's pathogenicity (FLB < 8, Jarvik and Browning 2016). However, this conclusion is highly sensitive to the parameter values (Fig. 1B, left). In particular, reducing the carriers' risk amplifies the evidence, a consequence of the unaffected carriers.

In order to better capture the apparent late-onset nature of the disease in this family, a possible approach is to introduce age-specific liability classes. Yet, with limited disease knowledge, this would require speculative estimates of the penetrance values. Furthermore, as each liability class adds multiple new parameters, sensitivity analyses become increasingly unwieldy.

Instead, we use the *relative risk* mode of shinyseg, as it streamlines this process while ensuring transparent assumptions. For instance, using the same risk values as before and an age of onset for phenocopies of 60 ± 15 years, we obtain an FLB score of 30.3 under a proportional hazards model, i.e. constant relative risk. This indicates that our data actually provides moderate evidence for pathogenicity (FLB > 16), a conclusion which is highly robust to the lifetime risk parameters (Fig. 1B, right).

## 4 Conclusion

We present shinyseg, an app for clinical cosegregation analysis that emphasizes flexible penetrance specification and sensitivity analysis. Suited to varying levels of expertise, our app encourages transparent modelling assumptions and guides the user toward well-informed decisions.

shinyseg is freely available at https://chrcarrizosa.shinyapps.io/shinyseg, and runs in all common browsers. Documentation and complete source code can be found on https://chrcarrizosa.github.io/shinyseg and https://github.com/chrcarrizosa/shinyseg.

## Supplementary Material

btae201_Supplementary_Data

## Data Availability

All code and data are accessible at https://github.com/chrcarrizosa/shinyseg.
