# Supplementary material for: shinyseg: a web application for flexible cosegregation and sensitivity analysis
Source: Bioinformatics. 2024 Apr 10;40(5):btae201. doi: 10.1093/bioinformatics/btae201 (PMC11069105; doi:10.1093/bioinformatics/btae201)
Supplement: btae201_Supplementary_Data [file btae201_supplementary_data.pdf]

shinyseg: a web application for flexible cosegregation and sensitivity analysis

Christian Carrizosa, Dag E. Undlien, Magnus D. Vigeland

1 Software comparison

This section presents a brief overview and comparison of current cosegregation analysis tools. We focus on software providing readily available output for genetic variant clinical interpretation, namely the full-likelihood Bayes factor (FLB) (Thompson *et al.* 2003) or the cosegregation likelihood ratio (CSLR) (Mohammadi *et al* 2009). This includes the R packages `CoSeg` and `segregatr`, the web-server `COOL` v2, and the R Shiny app introduced in this work, `shinyseg`. The main characteristics of each are summarized in Table 1.

Table 1. A comparison of available cosegregation analysis tools for variant interpretation.

	`CoSeg`	`segregatr`	`COOL`	`shinyseg`
**General features**				
Interface	R	R	Website	R/Website
Output	CSLR	FLB	FLB	FLB
Open source	✓	✓	-	✓
Interactive GUI	-	-	Basic	Advanced
**Analysis support**				
Inheritance models	Autosomal dominant	Autosomal, X-linked	Autosomal	Autosomal, X-linked
Multiple phenotypes	-	✓	✓	✓
Consanguinity	-	✓	Limited	✓
**Penetrance**				
Built-in estimates	Cancer	-	Cancer	-
Parametric specification	✓	-	-	✓
Liability classes	-	✓	✓	✓
Sensitivity analyses	-	-	-	✓

1.1 CoSeg

`CoSeg` (Ranola and Shirts 2016) is an R implementation of Mohammadi *et al.*'s (2009) algorithm for computing the CSLR. It is limited to autosomal dominant inheritance and pedigrees compatible with the method assumptions, including absence of loops/inbreeding. Furthermore, it can only handle one phenotype at a time and may be computationally prohibitive for large families (50+ members). The variant penetrances adhere to a normal model, with `CoSeg` providing estimates for a few common cancer genes.

1.2 segregatr

`segregatr` (Ratajska *et al.* 2023) is an R implementation of Thompson *et al.*'s (2003) approach for computing the FLB. Free from the constraints of `CoSeg`'s algorithm, `segregatr` is also more accessible thanks to its availability via CRAN. Together with `shinyseg`, it uniquely handles X-linked inheritance and most consanguineous cases, including pedigrees with parent-child matings. Moreover, it offers the most informative error reporting. In addition to the need to engage with R coding, `segregatr`'s main limitation is that it lacks guidance for defining the liability classes on which penetrance depends.

1.3 COOL

`COOL` v2 (Belman *et al.* 2020) is a website that also implements Thompson *et al.*'s (2003) method. As such, it shares several qualities with `segregatr`, while offering increased accessibility through a more convenient interface. `COOL`'s main forte lies in the inclusion of built-in cancer incidence data and even relative risk estimates for multiple genes, simplifying penetrance specification for those cases. For others, however, this step is less intuitive due to the lack of guidance, inaccurate error messages, and unavailable source code. `COOL`'s online-only nature may also pose challenges for clinical use in many countries.

1.4 shinyseg

`shinyseg` is an R Shiny app designed to address `segregatr`'s shortcomings by wrapping its features in a user-friendly interface. As a result, it is the most interactive tool, uniquely providing step-by-step feedback, real-time pedigree visualizations, and direction for the clinical interpretation of results. Additionally, `shinyseg` allows users to test cosegregation

assumptions through sensitivity analyses, and introduces a general and fully parametric version of `COOL`'s cancer penetrances. Its limitations are that it does not provide estimates for any particular disease or gene, and that sensitivity analyses can be slow.

2 Parametric penetrances

`shinyseg` offers different ways of defining the variant-disease model. For simple cases a manual specification of liability classes can suffice, but this becomes difficult when needing to account for onset age and multiple phenotypes. Here, we detail the parametric model that the app provides to streamline these more complex scenarios.

2.1 Overview

With its *relative risk* mode, `shinyseg` enables a parametric specification of the survival penetrances described in Belman *et al.* (2020). This model-based approach relies on two types of inputs for each disease phenotype d:

- The **baseline parameters** describe the incidence of d in non-carriers and heterozygous carriers in recessive inheritance. These include a baseline lifetime risk r_d^0, and the mean μ_d^0 and standard deviation σ_d^0 of their age of onset.
- The **hazard ratios** define the relative risk of d in homo-, hemi-, and heterozygous carriers in dominant inheritance, compared to the baseline. They can be constant or age-dependent, and may be specified either directly or through the variant-associated lifetime risk r_d^1.

Briefly, these parameters are used to calculate the baseline and variant-associated hazards for ages $t = 1, \ldots, 100$ years, and each phenotype d; subsequently, these hazards are used to derive the penetrances. The procedure is detailed in the following sections. Note that a sex-specific specification is also possible, in which case the computations are performed separately for each sex.

2.2 Baseline hazards

To define the baseline hazards $h^0_{d,t}$, we follow an approach akin to Jonker *et al.* (2003) and Mohammadi *et al.* (2009), using the cumulative distribution function (CDF) of a Normal distribution. One key difference is that `shinyseg` also incorporates truncation for greater control of the cumulative incidence within the parametric framework.

The relevant inputs are the baseline parameters: baseline lifetime risk r^0_d, mean μ^0_d and standard deviation σ^0_d of the age of onset. With these, the baseline cumulative incidences $CI^0_{d,t}$ are taken from the CDF of a truncated Normal (μ^0_d, σ^0_d) multiplied by r^0_d:

$$CI^0_{d,t} = \frac{\Phi\left(\frac{t-\mu^0_d}{\sigma^0_d}\right) - \Phi\left(\frac{0-\mu^0_d}{\sigma^0_d}\right)}{\Phi\left(\frac{100-\mu^0_d}{\sigma^0_d}\right) - \Phi\left(\frac{0-\mu^0_d}{\sigma^0_d}\right)} \cdot r^0_d,$$

where Φ is the CDF of a standard Normal. The truncation, at 0 and 100 years, ensures that the cumulative incidences at these time points equal 0 and r^0_d.

The baseline hazards $h^0_{d,t}$ are then derived as:

$$h^0_{d,t} = \log(1 - CI^0_{d,t-1}) - \log(1 - CI^0_{d,t})$$

2.3 Variant-associated hazards

The variant-associated hazards $h^1_{d,t}$ result from multiplying the baseline $h^0_{d,t}$ by the user-specified hazard ratios $\frac{h^1_{d,t}}{h^0_{d,t}}$. While this approach aligns with Belman *et al.* (2020), `shinyseg` introduces a distinctive model-based specification for these relative risks.

To elaborate, users provide a vector b_d as input, where values loosely represent the hazard ratios at equidistant ages from 1 to 100 years; this vector has a variable length H_d, allowing to specify a flexible number of age points. These values undergo a smoothing process across the entire age range based on B-splines, resulting in the age-specific ratios:

$$\frac{h^1_{d,t}}{h^0_{d,t}} = \exp\left(\sum_{j=1}^{H_d} \log b_{d,j} B_{d,j}(t)\right)$$

Here, $b_{d,j}$ and $B_{d,j}(t)$ refer to the j-th element of b_d and j-th basis function, respectively.

If a single value is provided, the basis function is set to 1, leading to a constant, i.e. age-independent, hazard ratio b_d.

These smoothed hazard ratios are then utilized to calculate $h^1_{d,t}$:

$$h^1_{d,t} = \frac{h^1_{d,t}}{h^0_{d,t}} \cdot h^0_{d,t}$$

2.3.1 Variant-associated lifetime risk

To facilitate hazard ratio specification and enable further sensitivity analyses, we provide the variant-associated lifetime risk r^1_d. This parameter represents the cumulative incidence of d in variant carriers (homo-, hemi-, and heterozygous carriers in dominant inheritance) at 100 years of age, and it is dynamically updated based on the other inputs.

More importantly, it can also be directly modified, resulting in the scaling of the specified hazard ratios to align with the new value. In practical terms, this allows users to use the hazard ratio input b_d to define a relative risk 'pattern'—whether constant or age-dependent—and subsequently adjust r^1_d to tailor it to the desired lifetime risk. Internally, this involves optimizing a scaling factor λ_d to minimize the expression:

$$\log\left(1 - r^1_d\right) + \sum_{t=1}^{100} \left(\exp\left(\sum_{j=1}^{H_d} \lambda_d \log b_{d,j} B_{d,j}(t) \right) \cdot h^0_{d,t} \right),$$

which is achieved using a quasi-Newton algorithm via the `optim()` `R` function.

2.4 Survival penetrances

The last step involves calculating the penetrances following a survival model.

First, the overall baseline and variant-associated cumulative incidences, CI^0_t and CI^1_t, are computed by treating the D disease phenotypes as independent and summing their individual contributions:

$$CI^0_t = 1 - \exp\left(- \sum_{d=1}^{D} \sum_{i=1}^{t} h^0_{d,i} \right) \qquad CI^1_t = 1 - \exp\left(- \sum_{d=1}^{D} \sum_{i=1}^{t} h^1_{d,i} \right)$$

The baseline and variant-associated (survival) penetrances, denoted as $SP^0_{d,t}$ and $SP^1_{d,t}$, are finally calculated as described in Belman *et al.* (2020):

$$SP^0_{d,t} = \begin{cases} \text{affected: } (1 - CI^0_{t-1}) \cdot h^0_{d,t} \\ \text{unaffected: } CI^0_t \end{cases} \qquad SP^1_{d,t} = \begin{cases} \text{affected: } (1 - CI^1_{t-1}) \cdot h^1_{d,t} \\ \text{unaffected: } CI^1_t \end{cases}$$

References

Belman S, Parsons MT, Spurdle AB *et al.* Considerations in assessing germline variant pathogenicity using cosegregation analysis, *Genet Med 2020*, **22**:2052–59.

Jonker MA, Jacobi CE, Hoogendoorn WE *et al.* Modeling familial clustered breast cancer using published data, *Cancer Epidemiol Biomarkers Prev 2003*, **12**:1479–85.

Mohammadi L, Vreeswijk MP, Oldenburg R *et al.* A simple method for co-segregation analysis to evaluate the pathogenicity of unclassified variants; BRCA1 and BRCA2 as an example, *BMC Cancer 2009*, **9**:211.

Ranola J and Shirts B. CoSeg R Package, 2016, R package version 0.55, <https://r-forge.r-project.org/projects/coseg/>.

Ratajska A, Vigeland MD, Wirgenes KV *et al.* The use of segregation analysis in interpretation of sequence variants in SMAD3: A case report, *Mol Genet Genomic Med 2023*; **11**:e2107.

Thompson D, Easton DF, and Goldgar DE. A full-likelihood method for the evaluation of causality of sequence variants from family data, *Am J Hum Genet 2003*; **73**:652-5.
